# Supplementary material for: Genetic evidence further elucidates the history and extent of badger introductions from Great Britain into Ireland
Source: R Soc Open Sci. 2020 Apr 1;7(4):200288. doi: 10.1098/rsos.200288 (PMC7211870; doi:10.1098/rsos.200288)
Supplement: Additional Figures relating to analyses described in results section of main text and supplementary materials [file rsos200288supp1.docx]

**Genetic evidence further elucidates the history and extent of badger introductions from Great Britain into Ireland.**

Adrian Allen^1^*, Jimena Guerrero^2^, Andrew Byrne^1^, John Lavery^1^, Eleanor Presho^1^, Emily Courcier^3^, James O’Keeffe^4^, Ursula Fogarty^5^, Richard Delahay^6^, Gavin Wilson^7^, Chris Newman^8^, Christina Buesching ^8^, Matthew Silk^9^, Denise O’Meara^10^, Robin Skuce^1^, Roman Biek^11^, Robbie A. McDonald^9^

^1^ Agri-Food and Biosciences Institute, Belfast, UK.

^2^ Centre D’Ecologie Fonctionelle et Evolutive , Montpellier, France.

^3^ Department of Agriculture, Environment and Rural Affairs, Belfast, UK.

^4^ Department of Agriculture Food and the Marine, Ireland.

^5^ Irish Equine Centre, County Kildare, Ireland.

^6^ Animal and Plant Health Agency, UK.

^7^ Biocensus Ltd, Gloucestershire, UK

^8^ Wildlife Conservation Research Unit, University of Oxford, UK.

^9^ Environment and Sustainability Institute, University of Exeter, Penryn, UK.

^10^ Waterford Institute of Technology, Ireland.

^11^ University of Glasgow, UK.

*Corresponding author


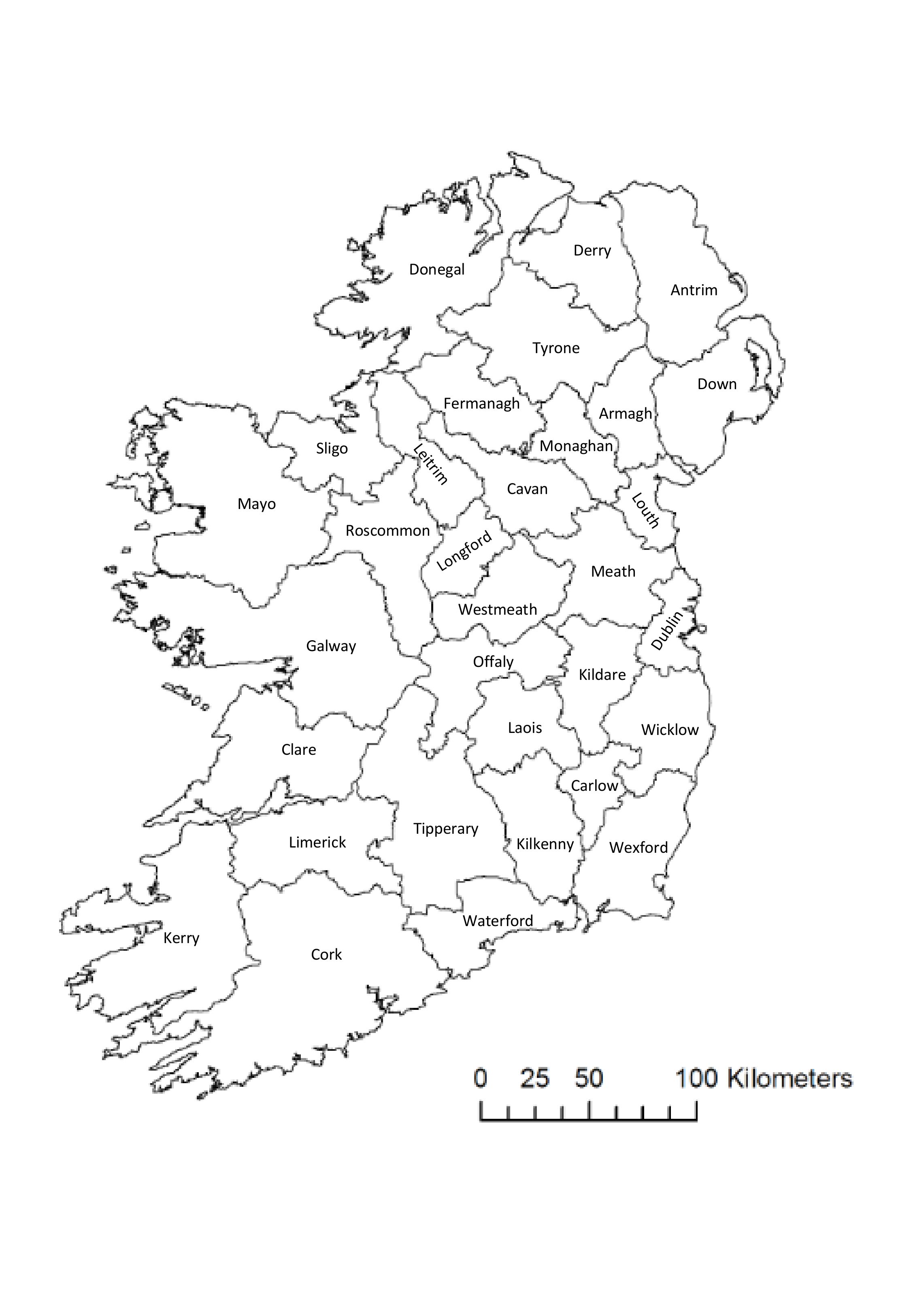


**Supplementary Figure S1** – Map of Ireland with County names and boundaries.


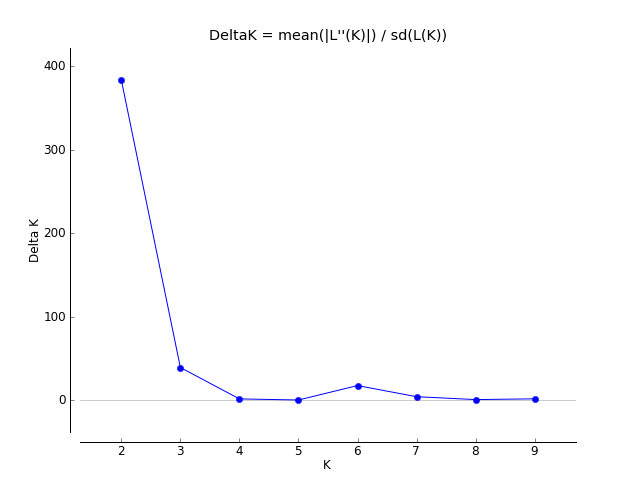


**Figure S2 -** STRUCTURE correlated allele frequencies model output. Evanno ΔK method plot of rate of change of log probability between consecutive K values.High rates of change of log probability at K=2 are indicative of hierarchical clustering in this data-set, indicative of two distinct genetic clusters.


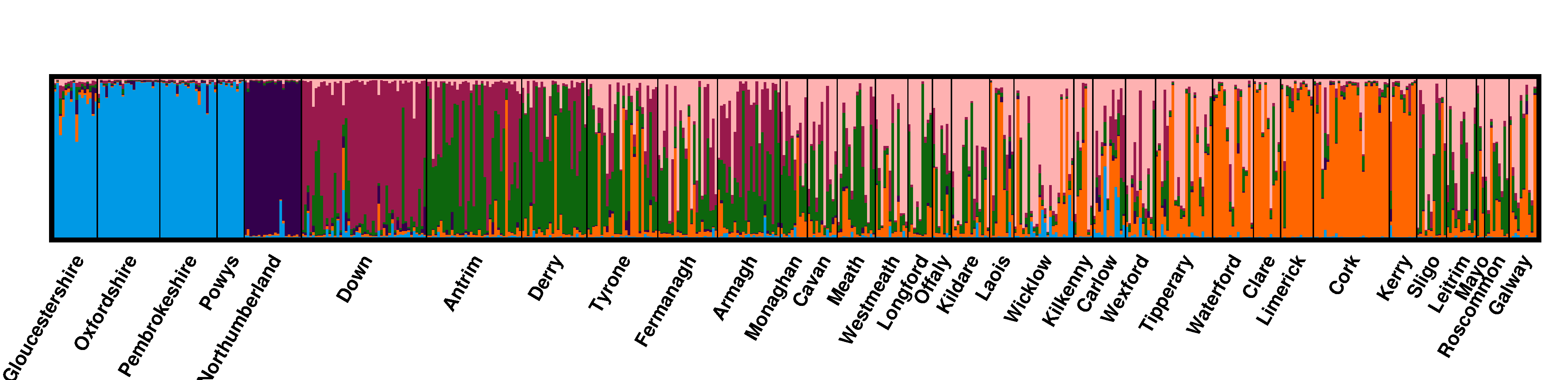


**Figure S3 -** STRUCTURE microsatellite admixture bar-plot K=6 for all badgers.


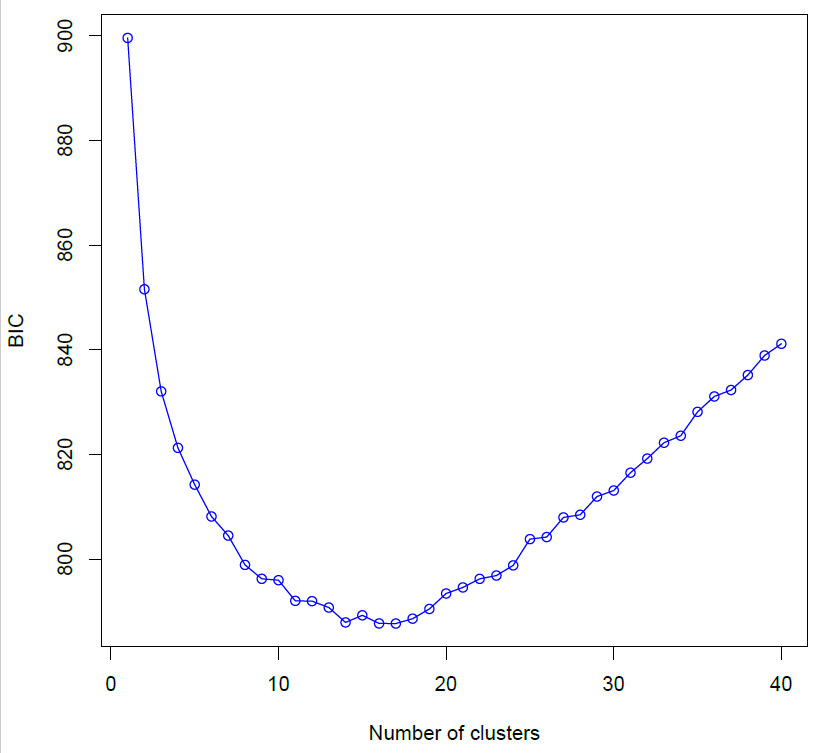


**Figure S4** – adegenet *find.clusters* function output when applied to all 545 GB and Irish badgers. Most likely value of K is indicated by point at which curve begins to plateau and reaches lowest value of Bayesian Information Criterion (BIC). In this case, K=14 clusters.


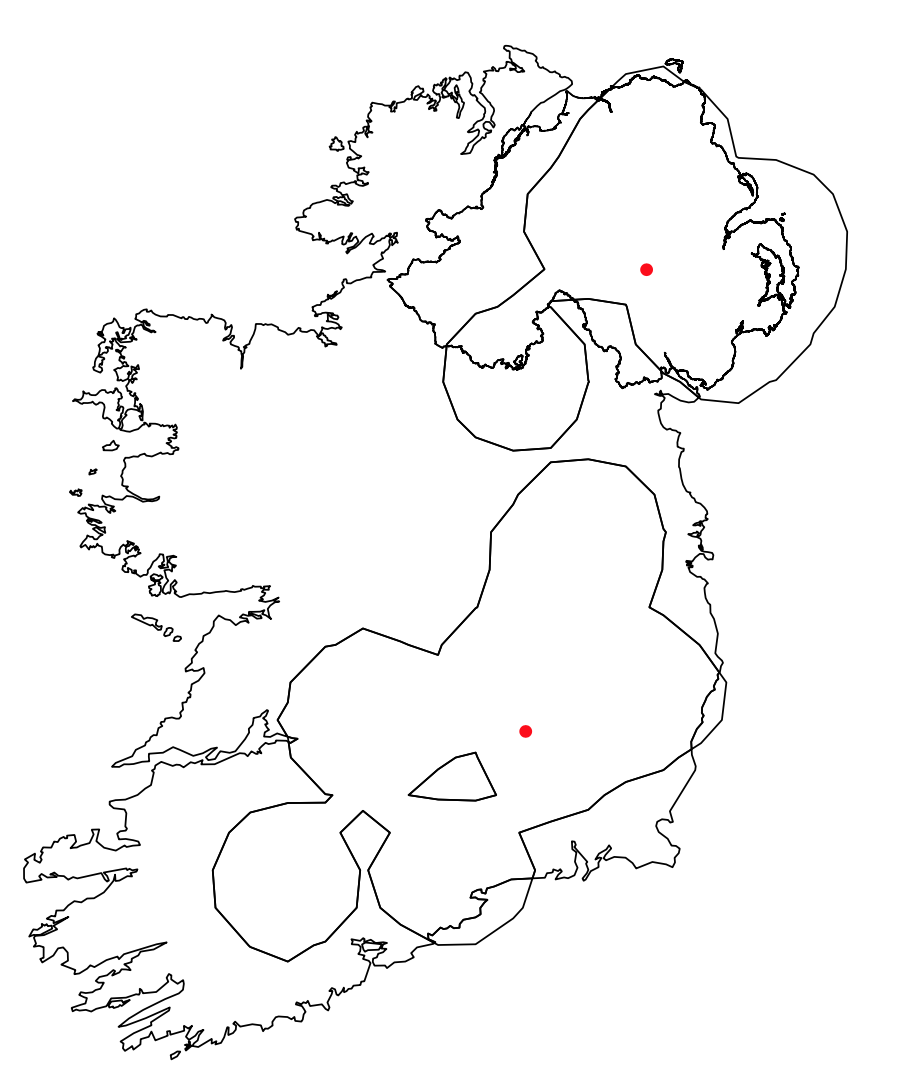


**Figure S5 -** 95% Kernel with highlighted spatial points centroid in red, for GB associated haplotypes H4 and H1.

**
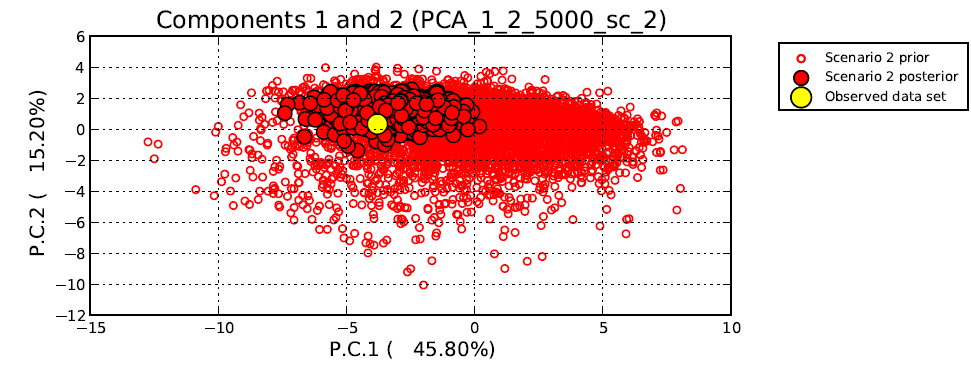
**

**Figure S6** – Do It Yourself Approximate Bayesian Computation (DIYABC) Principle Components Analysis (PCA). Model checking of GB and Ireland badger admixture scenario 2 by PCA - Simulated prior and posterior dataset summary statistics (mean number of alleles and mean genic diversity, Garza and Williamson’s M-ratio, F_st_ and δμ^2^ distance) for historical scenario 2 are compared to observed data set summary statistics. Nesting of observed summary statistics within prior and posterior simulated summary statistics derived from historical scenario 2 parameters, indicates the historical model explains the observed data.
