# Supplementary material for: Genetic evidence further elucidates the history and extent of badger introductions from Great Britain into Ireland
Source: R Soc Open Sci. 2020 Apr 1;7(4):200288. doi: 10.1098/rsos.200288 (PMC7211870; doi:10.1098/rsos.200288)
Supplement: Tables referred to in main text [file rsos200288supp2.docx]

**Genetic evidence further elucidates the history and extent of badger introductions from Great Britain into Ireland.**

Adrian Allen^1^*, Jimena Guerrero^2^, Andrew Byrne^1^, John Lavery^1^, Eleanor Presho^1^, Emily Courcier^3^, James O’Keeffe^4^, Ursula Fogarty^5^, Richard Delahay^6^, Gavin Wilson^7^, Chris Newman^8^, Christina Buesching ^8^, Matthew Silk^9^, Denise O’Meara^10^, Robin Skuce^1^, Roman Biek^11^, Robbie A. McDonald^9^

^1^ Agri-Food and Biosciences Institute, Belfast, UK.

^2^ Centre D’Ecologie Fonctionelle et Evolutive , Montpellier, France.

^3^ Department of Agriculture, Environment and Rural Affairs, Belfast, UK.

^4^ Department of Agriculture Food and the Marine, Ireland.

^5^ Irish Equine Centre, County Kildare, Ireland.

^6^ Animal and Plant Health Agency, UK.

^7^ Biocensus Ltd, Gloucestershire, UK

^8^ Wildlife Conservation Research Unit, University of Oxford, UK.

^9^ Environment and Sustainability Institute, University of Exeter, Penryn, UK.

^10^ Waterford Institute of Technology, Ireland.

^11^ University of Glasgow, UK.

*Corresponding author

| **Territory** | **County / Region** | **No. of badgers sampled** |
| --- | --- | --- |
| **Great Britain**  (n = 91) | Gloucestershire | 16 |
|  | Northumberland | 21 |
|  | Oxfordshire | 23 |
|  | Pembrokeshire | 21 |
|  | Powys | 10 |
| **Ireland**  (n = 454) | Antrim | 35 |
|  | Armagh | 23 |
|  | Carlow | 12 |
|  | Cavan | 11 |
|  | Clare | 10 |
|  | Cork | 28 |
|  | Derry | 24 |
|  | Down | 46 |
|  | Fermanagh | 22 |
|  | Galway | 10 |
|  | Kerry | 10 |
|  | Kildare | 14 |
|  | Kilkenny | 7 |
|  | Laois | 9 |
|  | Leitrim | 11 |
|  | Limerick | 12 |
|  | Longford | 9 |
|  | Mayo | 3 |
|  | Meath | 14 |
|  | Monaghan | 10 |
|  | Offaly | 7 |
|  | Roscommon | 9 |
|  | Sligo | 11 |
|  | Tipperary | 21 |
|  | Tyrone | 26 |
|  | Waterford | 15 |
|  | Westmeath | 12 |
|  | Wexford | 11 |
|  | Wicklow | 22 |

**Table S1** – Numbers of badger samples submitted per region across Ireland and Great Britain.

| **Structure Model** | **Mean log likelihood of K value (LnP(K)) for 2 genetic clusters (K=2)** | **Mean log likelihood of K value (LnP(K)) for 6 genetic clusters (K=66)** |
| --- | --- | --- |
| **Independent allele frequencies** | -16874.37 | -16101.86 |
| **Correlated allele frequencies** | -16849.34 | -15932.88 |

**Table S2** – Mean log likelihood of K values from Structure Harvester outputs for both the independent and correlated allele frequency models applied to all badger data.

|  | **Gloucestershire** | **Oxfordshire** | **Pembrokeshire** | **Powys** | **Northumberland** | **Irish 1** | **Irish 2** |
| --- | --- | --- | --- | --- | --- | --- | --- |
| **Gloucestershire** | 0 |  |  |  |  |  |  |
| **Oxfordshire** | 0.14* / 0.23 | 0 |  |  |  |  |  |
| **Pembrokeshire** | 0.18* / 0.32 | 0.14* /0.20 | 0 |  |  |  |  |
| **Powys** | 0.19* / 0.36 | 0.18* / 0.26 | 0.20* / 0.29 | 0 |  |  |  |
| **Northumberland** | 0.19* / 0.37 | 0.22* / 0.36 | 0.21* / 0.34 | 0.27* / 0.49 | 0 |  |  |
| **Irish 1** | 0.21* / 0.37 | 0.26* / 0.43 | 0.26* / 0.42 | 0.32* / 0.56 | 0.22* / 0.35 | 0 |  |
| **Irish 2** | 0.15* / 0.33 | 0.18* / 0.32 | 0.16* / 0.27 | 0.21* / 0.39 | 0.15* / 0.27 | 0.05* /0.08 | 0 |

**Table S3** - Pairwise F_st_ and Jost’s D between the five regional British sub-populations and Irish sub-populations 1 and 2. For F_st_, significance was tested by 420 permutations in FSTAT * = p<0.05
